# Identifying lumbosacral plexus nerve root abnormalities in patients with sciatica using 3T readout-segmented echo-planar diffusion weighted MR neurography

**DOI:** 10.1186/s13244-021-00992-w

**Published:** 2021-04-20

**Authors:** Osamah M. Abdulaal, Allison McGee, Louise Rainford, Dearbhail O’Driscoll, Marie Galligan, Valerie Reid, Peter J. MacMahon

**Affiliations:** 1 Diagnostic Radiology Technology, College of Applied Medical Sciences, Taibah University, Madina, Saudi Arabia; 2 Radiography and Diagnostic Imaging, School of Medicine, University College Dublin, Dublin, Ireland; 3 Department of Radiology, Mater Misericordiae University Hospital, Eccles Street, Dublin7, Dublin, Ireland; 4 School of Medicine, University College Dublin, Dublin 4, Ireland; 5 Department of Neurophysiology, Mater Misericordiae University Hospital, Eccles Street, Dublin7, Dublin, Ireland

**Keywords:** Magnetic resonance imaging, Evidence-based practice, Spine, Sciatica

## Abstract

**Objectives:**

To investigate the accuracy of Diffusion Weighted Imaging (DWI) using the Readout Segmentation of Long Variable Echo-trains (RESOLVE) sequence in detecting lumbosacral nerve abnormalities.

**Methods:**

Following institutional ethics committee approval, patients with sciatica-type lower limb radicular symptoms (*n* = 110) were recruited and prospectively scanned using 3T MRI. Additional participants (*n* = 17) who underwent neurophysiological testing (EMG/NCV), were also prospectively studied. In addition to routine lumbar spine MRI, a DWI-RESOLVE sequence of the lumbosacral plexus was performed. Two radiologists, blinded to the side of patient symptoms, independently evaluated the MR images. The size and signal intensity changes of the nerves were evaluated using ordinal 4-point Likert-scales. Signal-to-noise ratio (SNR), apparent diffusion coefficient (ADC) and size were measured for affected and normal nerves. Inter-observer agreement was determined with kappa statistics; κ.

**Results:**

In patients who did not undergo EMG/NCV testing (*n* = 110), the DWI-RESOLVE sequence detected lumbosacral nerve abnormalities that correlated with symptoms in 36.3% (40/110). This is a similar percentage to patients who underwent EMG/NCV testing, which was positive and correlated with symptoms in 41.2% (7/17). Inter-observer agreement for evaluation of lumbosacral nerve abnormalities was excellent and ranged from 0.87 to 0.94. SNR and nerve size measurements demonstrated statistically significant differences for the L5 and S1 nerves (*p* value < 0.05) for patients who did not undergo EMG/NCV testing.

**Conclusion:**

The DWI-RESOLVE sequence is a promising new method that may permit accurate detection and localization of lumbar nerve abnormalities in patients with sciatica.

## Key points

- DWI-RESOLVE appears to accurately detect lumbosacral nerve abnormalities.
- The DWI-RESOLVE MR sequence has potential value in objectively confirming a neural cause for radicular symptoms, localizing the cause of symptoms and assessing response to therapies.
- The DWI-RESOLVE sequence, which requires no ionizing radiation or contrast administration, successfully detected all lumbosacral nerve abnormalities determined by EMG/NCV.

## Introduction

Sciatica, which refers to pain that conforms to the sciatic nerve distribution, is a common cause of disability worldwide [1]. Identifying the cause of sciatica-type symptoms can be problematic. The majority of patients with sciatica have a herniated intervertebral disc on magnetic resonance imaging (MRI); however, it may not be clear which disc level is symptomatic [2, 3]. Furthermore, sciatica-type symptoms may be non-discogenic in origin. Symptoms related to tumors, fracture, synovial cysts, sacroiliitis, hip joint pathology, gluteal tendinopathy and other conditions may be confused with true sciatica [4]. Radiculopathy symptoms can be investigated using electromyography and nerve conduction velocity (EMG/NCV) testing; however, these examinations are invasive with limited sensitivity [5, 6].

MRI is employed extensively to evaluate the lumbosacral spine in patients with radiculopathy, with T2-/T1-weighted Turbo Spin Echo (TSE) and T2-weighted fat suppression techniques currently the most common sequences included in lumbar spine MRI protocols [7]. These sequences are generally excellent at assessing for anatomical abnormalities and some physiological changes such as bone marrow edema, but not as useful at directly detecting abnormalities in nerve roots. Standard TSE MRI is recognized as being insufficient for the evaluation of the lumbosacral nerves, mainly due to poor contrast between nerves and adjacent structures [8, 9].

MR neurography techniques are utilized in some centers to identify abnormalities of the nerve roots and lumbosacral plexus more directly [10]. Diffusion weighted imaging (DWI) is one such technique used in MR neurography to evaluate the lumbosacral nerves [7, 8, 11–15]. Recently, a DWI sequence, known as Readout Segmentation of Long Variable Echo-trains (RESOLVE), a vendor-specific sequence, has become available in routine clinical practice with the increasing availability of 3T scanners [16–18]. The advantages of this sequence are potentially high-quality, high-resolution DWI images by reducing susceptibility artifacts, distortion and blurring relative to single shot echo planar-based DW imaging (SS-EPI) [16, 17].

While MR neurography has many potential advantages, it is not a routine component of a lumbar spine MRI protocol. We investigated a tailored DWI-RESOLVE sequence for the lumbosacral plexus, that would not add excessive time to a typical lumbar spine MRI examination, but would relatively clearly delineate the lumbosacral nerves and abnormalities of the plexus. Such a sequence may be generally applicable to daily practice and potentially interpretable by non-experts. The aim of this study was to investigate the accuracy of the DWI-RESOLVE sequence in detecting lumbosacral plexus nerve root abnormalities.

## Materials and methods

Institutional ethical approval was granted for this study. Written informed consent was obtained from all subjects before the examination. Over a six-month period, all adult patients referred for lumbar spine MRI by Orthopedic and Neurology Departments with symptoms suggestive of unilateral radiculopathy (i.e., unilateral pain radiating from the lumbar spine towards the leg) were considered for inclusion. Together with the available information in the referrals, sciatica bothersomeness index, which includes back pain, leg pain, numbness and leg weakness [19] was also used for recruitment purposes. For optimum results, all patients were checked to ensure that they were symptomatic at the time of the MRI examination. Patients with any contraindication to MRI, including those with a lumbar spinal stabilization implant, were excluded from the study. In total, 110 patients with radiculopathy were prospectively included (female *n* = 67; male *n* = 43; age range: 22–68 years; mean age: 48 years).

An additional 17 symptomatic patients (female *n* = 11; male *n* = 6; age range: 37–69 years; mean age: 54 years) who had EMG/NCV testing for sciatica symptoms were also prospectively recruited.

### MR imaging protocol

Lumbosacral spine imaging was undertaken on a 3T whole-body MR system (MAGNETOM *Skyra*, Siemens Healthcare GmbH, Erlangen, Germany) with a 32-channel spinal phased array coil used together with an 18-channel body-matrix coil placed over the patient’s lower abdomen/pelvis.

All patients underwent both a routine 2D lumbar spine MR scanning protocol and the axial DWI-RESOLVE sequence (Table 1) in a single scanning session. The high-resolution DWI-RESOLVE sequence included the following* b* values 50, 500 and 800 s/mm^2^, together with Spectral Attenuated Inversion Recovery (SPAIR) fat suppression technique.

**Table 1:** Sequence parameters for standard MRI lumbar spine protocol and DWI-RESOLVE

Parameters	DWI-RESOLVE (*b* values: 50; 500; 800)	Sagittal T2 TSE	Sagittal T1 TSE	Axial T2 TSE
TR / TE (ms)	12,700 / 57	3500/92	650/8.6	2870/106
ETL	NA	17	3	21
FOV read (mm^2^)	250	280	280	190
Voxel size	1.9 × 1.9x4	0.7 × 0.7x4	0.4 × 0.4x4	0.6 × 0.6x4
Average	1	1	1	2
Bandwidth (Hz/px)	1012	250	252	250
Flip angle (°)	180	160	150	160
Acceleration factor	2	2	None	2
TA (minutes)	10:11	2:10	3:01	1:06

TR = repetition time; TE = echo time; ETL = echo train length; FOV = field of view; TA = acquisition time

In an attempt to better demonstrate abnormal nerves on the DWI sequence, color overlays in the region of the plexus were created that highlighted pixels where DWI signal was relatively high on both b-500 and b-800 images. Initial post-processing was performed using MANGO (Multi-Image Analysis GUI) software (Research Imaging Institute, University of Texas Health Science Center, San Antonio, TX. www.ric.uthscsa.edu/mango). In this software, b-500 and b-800 images for each patient were correlated anatomically and an image calculation performed (the difference between the b-500 and b-800 pixel values was added to the b-500 pixel value). This new image was subsequently imported into a separate software program, Osirix *PRO* (Osirix *PRO*, Aycan Medical Systems, NY, USA). An oval ROI was manually placed to encompass the lumbosacral plexus on this imported dataset and propagated across the series. All pixels outside the ROI were set to zero to reduce the conspicuity of pixel changes related to bowel content introduced by the post-processing technique. This oval-shaped post-processed series is then color-overlaid onto the original b-800 series and fused.

### Image analysis

#### Qualitative evaluation

Evaluation was performed by two musculoskeletal (MSK) radiologists, with 8 and 10 years of experience, respectively using a PACS monitor with 5-megapixel resolution (*Barco™*). One radiologist evaluated the standard lumbosacral 2D TSE images. Both readers independently evaluated the images acquired using the DWI-RESOLVE sequence. The radiologists were blinded to each patient’s clinical history and clinical findings.

Initially, radiologists were educated on normal plexus imaging obtained from asymptomatic volunteers. In addition, example cases demonstrating the various grades of nerve abnormalities, as outlined below, were presented to the radiologists. The radiologists graded the size and signal intensity changes of lumbosacral nerves (L5, S1, S2, S3, and sciatic nerves) in the study population by using ordinal 4-point Likert-scales. The radiologists could refer to the education material at any time and were also instructed to use normal-appearing nerves identified during the study as a comparison when determining the grade of abnormalities. Nerve size evaluation was rated as follows: normal size (Grade I); nerve less than 50% larger than normal (Grade II); nerve 50–100% larger (Grade III); nerve more than 100% larger (Grade IV) [20]. Similarly, the evaluation of the signal intensity was rated as follows: isointense (Grade I); nerve mildly hyperintense relative to normal (Grade II); nerve moderately hyperintense (Grade III); nerve markedly hyperintense (Grade IV) [20].

#### Quantitative evaluation

**Fig. 1:**
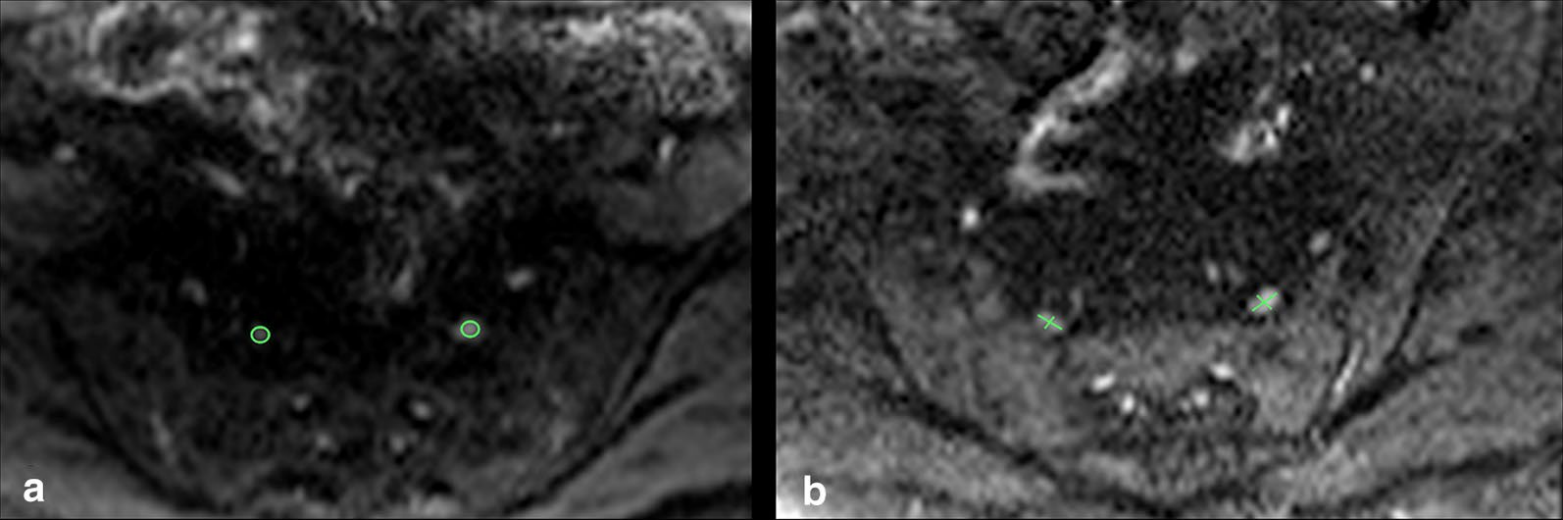
DWI-RESOLVE images on two different patients demonstrating typical nerve region of interest (ROI) placements (**a**) and dimensional calipers (**b**) for quantitative measurements

Using *Syngo* software (*Syngo*, Siemens Medical Solutions, Erlangen, Germany), a specialist MR radiographer measured the signal-to-noise ratio (SNR), apparent diffusion coefficient (ADC) and size. The abnormal and normal nerves were evaluated by precisely drawing ROIs within the center of the nerves on axial DWI-RESOLVE images (Fig. 1). ROIs for each abnormal nerve (abnormal nerves agreed upon by the panel of two radiologists) were placed where the nerve appeared greatest in cross-sectional diameter and signal intensity (Fig. 1). This tended to be approximately 3–4 cm distal to the exit foramen. For each abnormal nerve, a ROI measurement was also obtained from the contralateral nerve at the same anatomical level. Occasionally, a slightly different level was picked to draw the ROIs for the contralateral side to avoid inaccurate measurements occurring from partial volume effect [7]. For SNR and ADC measurements, the ROIs were placed at the same spatial position for all* b* value images to avoid any bias. For size measurements, the anteroposterior (AP) and transverse dimensions of the nerves were assessed using the b-50 images (Fig. 1).

The SNR within the anatomical regions was calculated as follows [12]:

$${\text{SNR}} = {\text{SI}}_{{({\text{nerve}})}} /{\text{SD}}_{{({\text{noise}})}}$$

The SI _(nerve)_ is signal intensity of the nerves, and SD_(noise)_ is the standard deviation of the noise. The SD of noise was measured by placing circular ROI within the image background, avoiding artifact if present.

The ADC values were calculated using following formula [21]:

$$ADC \, = \, \left[ {{\text{In}}\;\left( {Sb0/Sb1} \right)} \right]/\left( {b1 - b0} \right)$$

(Sb0 = mean signal intensity for b-50 images, Sb1 = mean signal intensity for b-500 or b-800 images, *b*0 = 50, and *b*1 = 500 or 800).

## Reference standard

For patients on whom EMG/NCV testing was not performed (*n* = 110), the clinical symptoms/signs provided in the indication for the MRI were regarded as a surrogate standard to assess against DWI-RESOLVE findings. For patients who underwent EMG/NCV testing (*n* = 17), the clinical symptoms matched EMG/NCV findings in all cases.

### Statistical analysis

All statistical analyses were carried out using SPSS (IBM SPSS Statistics for Macintosh, Version 23.0, Armonk, NY: IBM Corp., 2015). Statistical significance was assumed for *p* < 0.05.

Kappa (*κ*) statistics with 95% confidence intervals determined the inter-observer agreement during MR image scoring. For each anatomical structure, SNR, ADC and size were compared using a Wilcoxon signed-rank test with Bonferroni correction. The SNR values for the abnormal and normal nerves were compared separately for each* b* value. The ADC values for the abnormal and normal nerves were compared for each ADC type. The size of the abnormal and the normal nerves was compared for each AP and transverse dimension as measured on axial DWI-RESOLVE images.

## Results

### Qualitative findings

During evaluation of the DWI-RESOLVE images acquired from (*n* = 127) patients, each radiologist qualitatively evaluated 1524 individual lumbosacral nerves (L4, L5, S1, S2, S3, and sciatic nerve). For patients on whom an EMG/NCV was not performed (*n* = 110), the MRI findings demonstrated that only 36.3% (40/110) of patients had findings on DWI-RESOLVE images consistent with a lumbosacral nerve abnormality. No abnormalities were recorded for the L4 and S3 nerves.

As agreed by both readers, 42 patients had lumbosacral nerve abnormality findings using DWI-RESOLVE, 40 of which matched the clinical indications (true positive). Both readers identified a total of 57 nerve abnormalities in the 42 patients; 5 of these abnormalities were found not to match the clinical indications and 52 nerve abnormalities were found to match the clinical indications.

The assigned nerve size and signal intensity grades were found to differ slightly between the two readers. Nerve size lumbosacral abnormalities recorded by reader 1 versus reader 2 were distributed as follows: Grade II = 27 vs 28 (reader 1 vs reader 2), Grade III = 21 vs 20, and Grade IV = 4 vs 4. The signal intensity changes reported by reader 1 versus reader 2 generated the following results: Grade II = 28 vs 26, Grade III = 20 vs 22, and Grade IV = 4 vs 4.

With regard to patients who also underwent EMG/NCV testing, 41.2% (7/17) of patients had lumbosacral nerve abnormality findings (7 nerve abnormalities) on DWI-RESOLVE, while 58.8% (10/17) were normal with no MR evidence of nerve abnormality. All the patients who had lumbosacral nerve abnormalities on DWI-RESOLVE matched both the clinical indications and the EMG/NCV findings. The DWI-RESOLVE sequence successfully detected all lumbosacral nerve abnormalities determined by EMG/NCV (7/7).

**Fig. 2.**
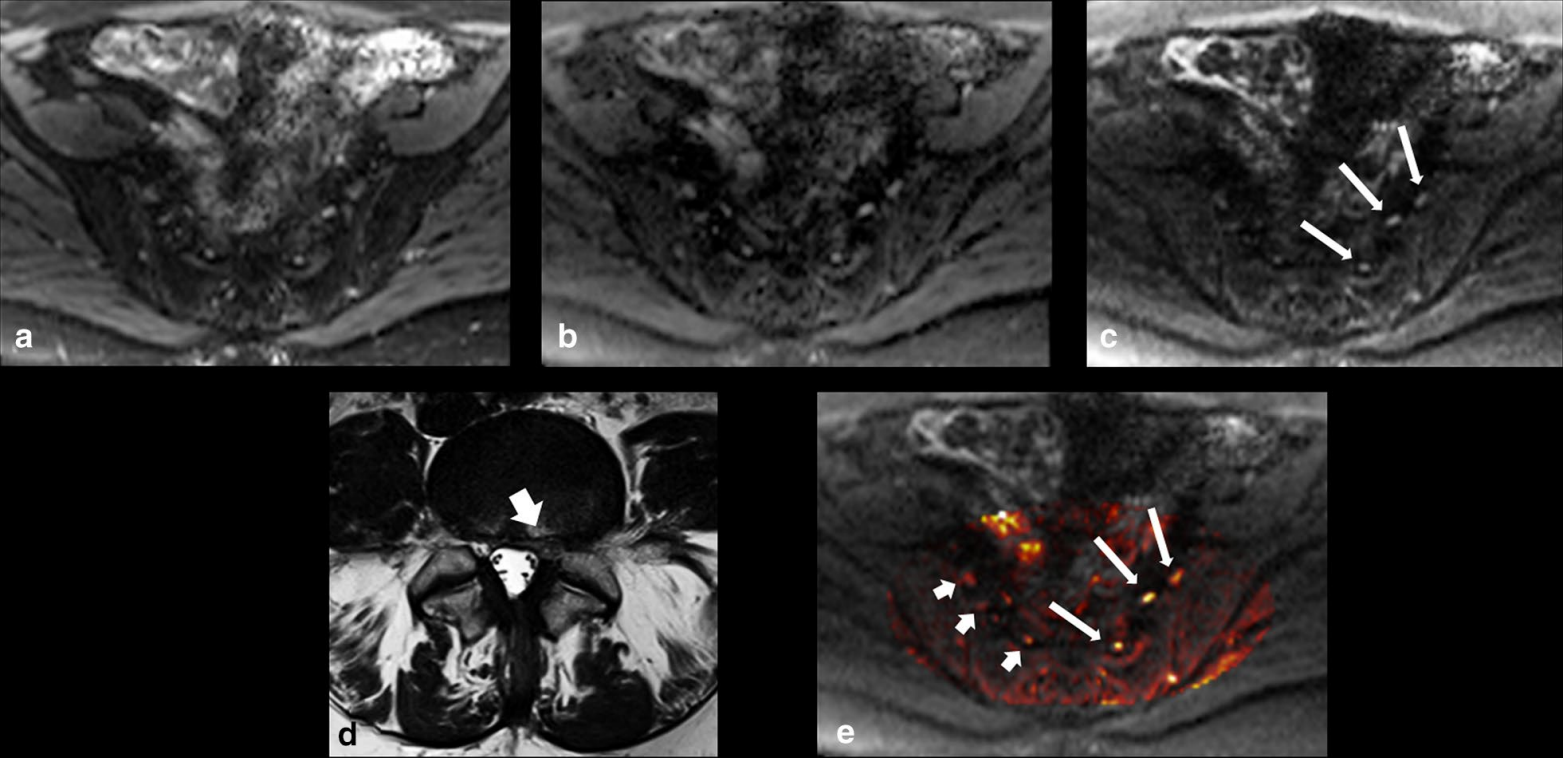
38-year-old female who presented with left leg pain and numbness. DWI-RESOLVE images (**a**) *b* = 50, (**b**) *b* = 500, (**c**) *b* = 800. The arrows on the b800 image correlate to the left L5, S1 and S2 nerves from anterior to posterior. The nerves were evaluated by both readers on axial DWI-RESOLVE images as positive for abnormality (grade II abnormal size and signal intensity). The left L5 nerve appears less involved compared to the left S1 and S2 nerve levels. Axial T2W TSE (**d**) of the lumbar spine at L5/S1 highlights a large left-sided disc herniation (arrow), which explains symptoms and correlates to nerve abnormalities. The color-mapped image **e** demonstrates the asymmetry of the lumbosacral plexus to better effect. Long arrows indicate abnormal left nerves and short arrows indicate normal contralateral side

**Fig. 3.**
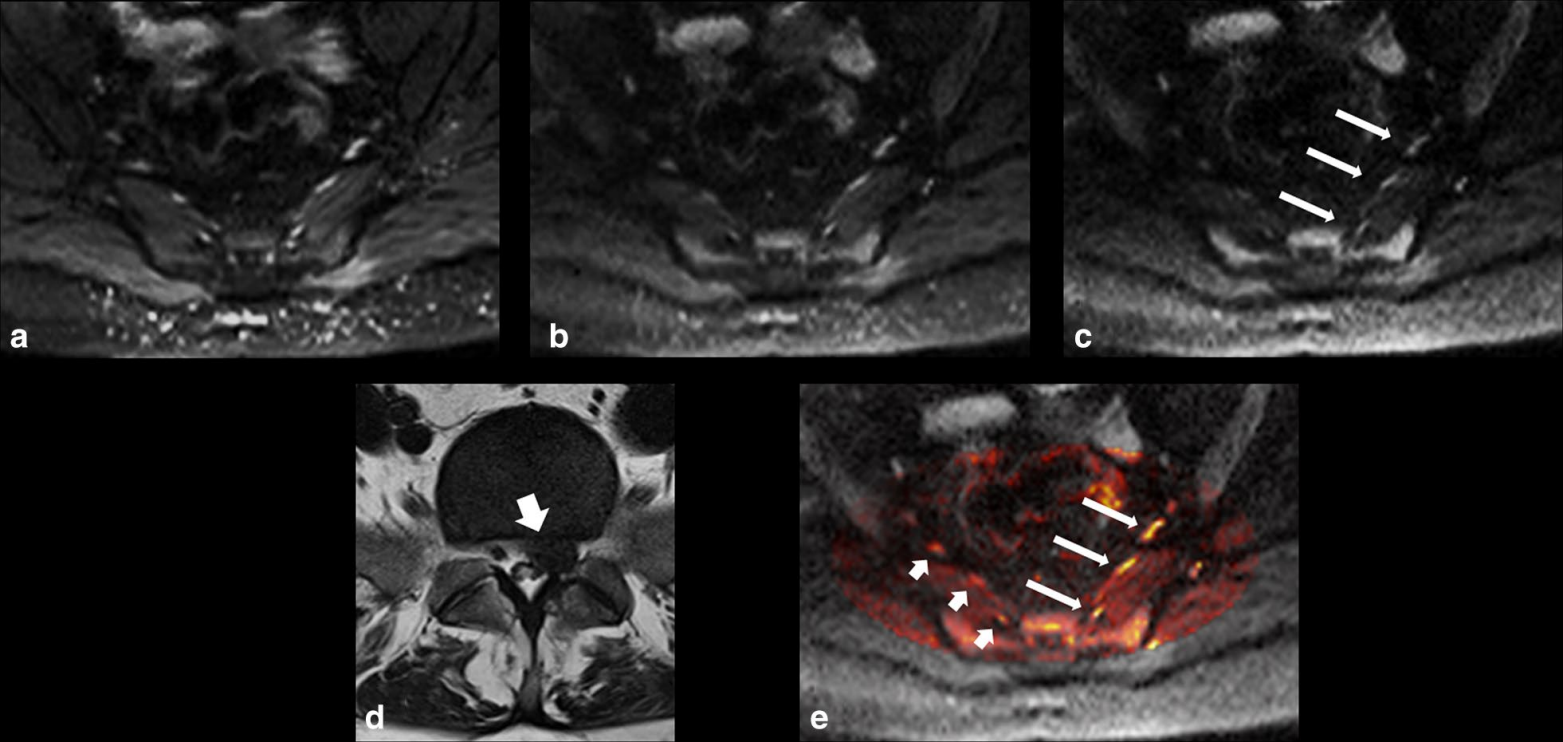
45-year-old female suffering from left leg pain, severe weakness and numbness. DWI-RESOLVE images (**a**) *b* = 50, (**b**) *b* = 500, (**c**) *b* = 800. The arrows on the b800 image correlate to the left L5, S1 and S2 nerves from anterior to posterior. The nerves were evaluated by both readers on axial DWI-RESOLVE images as positive for abnormality (grade III abnormal size and signal intensity). Axial T2W TSE **d** of the lumbar spine at L5/S1 highlights a large left-sided disc herniation (arrow), which explains symptoms and correlates to nerve abnormalities. The color-mapped image **e** demonstrates the asymmetry of the lumbosacral plexus to better effect in this patient. Long arrows indicate abnormal left nerves and short arrows indicate normal contralateral side

**Fig. 4.**
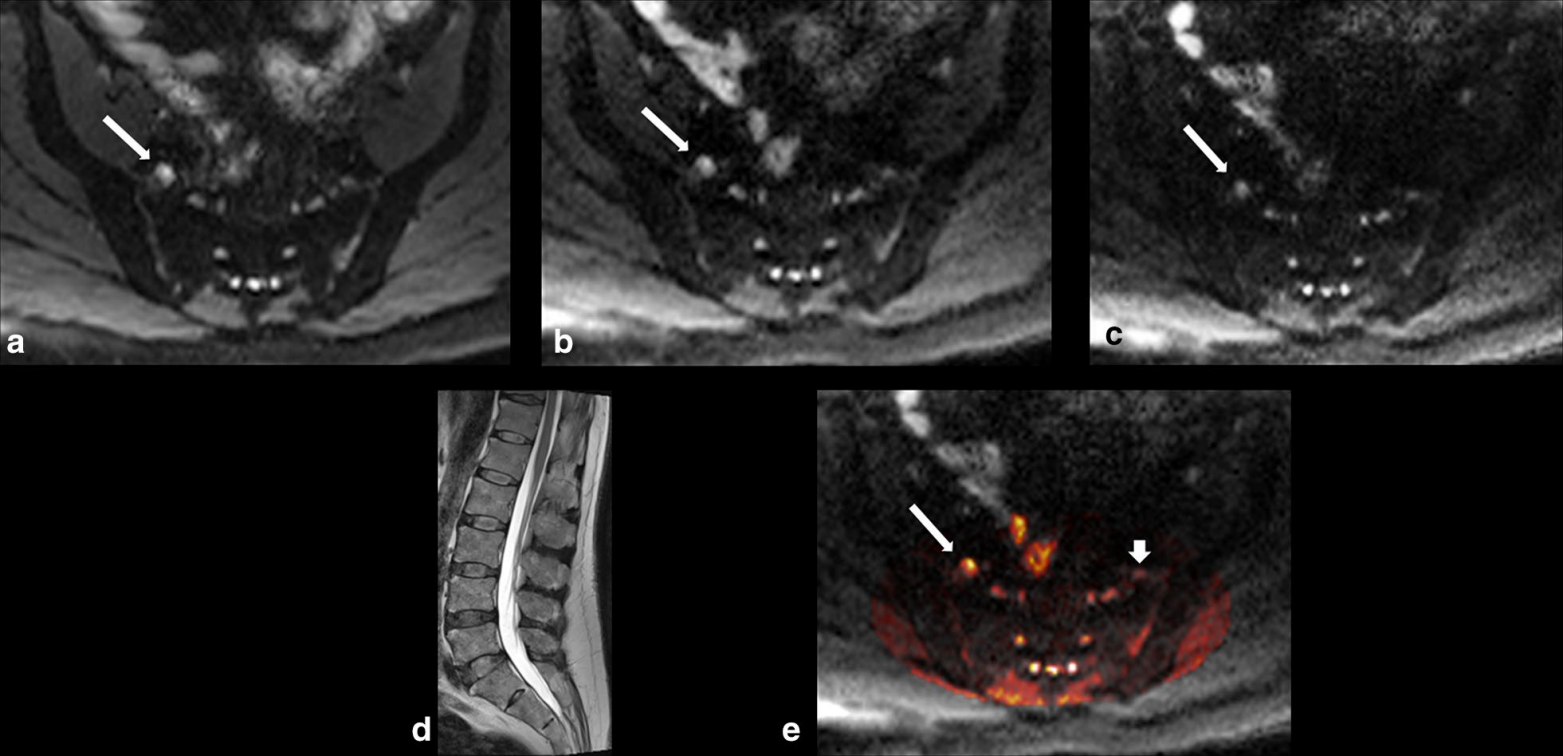
63-year-old man suffering from severe right leg pain and numbness. DWI-RESOLVE images **a** *b* = 50, **b** *b* = 500, **c** *b* = 800. The arrows on each of the* b* value images correlate to the right L5 nerve root. The nerve was evaluated by both readers on axial DWI-RESOLVE images as positive for abnormality (grade IV abnormal size and signal intensity). Sagittal T2W TSE (**d)** of the lumbar spine demonstrates no significant disc herniation or other potential cause for symptoms. The color-mapped image **e** demonstrates the asymmetry of the right L5 nerve. Long arrows indicate abnormal right L5 nerve and short arrow indicates normal contralateral left L5 nerve

For the 40 patients who had lumbosacral nerve abnormalities which matched their clinical indications and were agreed upon by both readers on DWI-RESOLVE, disc herniation and spinal stenosis were found to be the etiology in 87.5% (35/40) of cases based on appearances on the conventional 2D lumbar spine MR scanning protocol (Figs. 2 and 3). In 12.5% (5/40) of patients, there was no abnormality evident on the routine 2D T2W TSE spinal images; however, there were lumbosacral nerve abnormality findings on DWI-RESOLVE images (Fig. 4). Grade II, III and IV nerve abnormalities can be seen in Figs. 2, 3, and 4, respectively.

The inter-observer agreement κ for each nerve graded by the readers was calculated. The κ values revealed excellent inter-observer agreement for the evaluation of all lumbosacral nerve abnormalities for the DWI-RESOLVE sequence, ranging from 0.87 to 0.94. Inter-observer agreement values were 0.92 (95% CI: 0.87–0.97), 0.87 (95% CI: 0.75–0.99), 0.94 (95% CI: 0.85–1.0), and 0.93 (95% CI: 0.8–1.1) for L5, S1, S2, and the sciatic nerve, respectively.

In our study, readers found the color map images demonstrated abnormal nerves more favorably compared to the source DWI-RESOLVE images. The color map highlighted signal intensity increases with increasing* b* values in the region of interest (lumbosacral plexus). This improved the conspicuity of potentially abnormal nerves, and increased readers’ confidence.

**Fig. 5:**
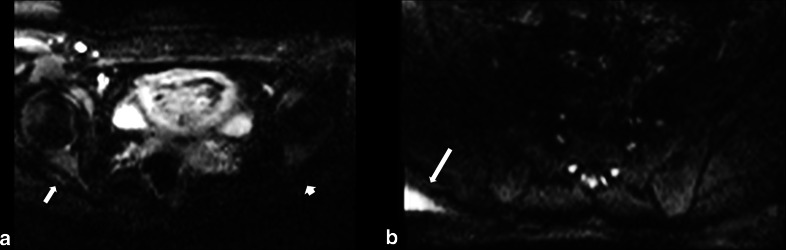
Demonstration of DWI-RESOLVE signal inhomogeneity. The b50 image (**a**) of a patient with normal neurophysiology testing demonstrates relatively prominent loss of signal on the left side of the body compared to the right. The left sciatic nerve (short arrow) is obscured due to this loss of signal. The normal right sciatic nerve (long arrow) appears asymmetric due to this spurious artifact. The b800 image (**b**) on a different patient demonstrates the hyperintense signal (arrow) that can occur at the edge of the field of view on some patients. This type of signal inhomogeneity does not typically obscure or distort the lumbosacral plexus

Susceptibility artifact adversely affected the visualization of the lumbosacral nerves located at the margins of the defined FOV in some (*n* = 8) cases (Fig. 5). Images from these patients were excluded from analysis in this study as the areas of susceptibility-related signal loss could make the lumbosacral nerves on the normal side appear artifactually more hyperintense than those on the contralateral side.

### Quantitative findings

The quantitative findings of this study for patients on whom an EMG/NCV was not performed (*n* = 110) included those patients (*n* = 40; 52 nerve abnormalities) with lumbosacral nerve abnormalities agreed upon by both readers on DWI-RESOLVE and found to match the clinical indications (true positive). In our study, SNR, ADC and size findings demonstrated higher mean values for the abnormal compared to normal nerves (Tables 2, 3 and 4). The values for mean SNR and size for the L5 and S1 nerves were significantly different (*p* < 0.05). However, there were no statistically significant differences in mean SNR or size for the S2 and sciatic nerves. This is likely due to the lower frequency of lumbar radiculopathy-related abnormalities for the S2 (*n* = 7) and sciatic (*n* = 4) nerves. The ADC findings did not differ significantly between the abnormal and normal nerves.

**Table 2:** Signal-to-noise ratio findings for lumbosacral nerves

Anatomical structures	Side	DWI-RESOLVE b50	DWI-RESOLVE b500	DWI-RESOLVE b800	
L5 nerve (*n* = 22)	Abnormal	42.7 (± 11.2)*	28.3 (± 10.6)*	21.9 (± 8.4)	
	Normal	30.1 (± 9.8)	21.6 (± 9.3)	16 (± 5.4)	
S1 nerve (*n* = 19)	Abnormal	53.4 (± 28.8)*	38.7 (± 18.5)*	30.2 (± 18)*	
	Normal	37.8 (± 20.5)	27 (± 15.6)	21.4 (± 16.4)	
S2 nerve (*n* = 7)	Abnormal	71.3 (± 33.3)	55.3 (± 34.3)	45.2 (± 32.1)	
	Normal	58.5 (± 27.9)	42 (± 24.8)	31.1 (± 20.9)	
Sciatic nerve (*n* = 4)	Abnormal	51.5 (± 7.9)	31.7 (± 5.1)	26.4 (± 2.6)	
	Normal	31.8 (± 3.6)	21.5 (± 4.4)	16.4 (± 1.5)	

Mean (± SD) of SNR shown by sequence type and anatomical structure, and *p* values from Wilcoxon signed-rank tests were corrected for 12 tests

*Bonferroni-adjusted *p* value < 0.05 when the abnormal nerve was compared to the normal nerve

**Table 3:** Apparent diffusion coefficient findings (× 10^–3^ mm^2^/s) for lumbosacral nerves

Anatomical structures	ADC type	Abnormal nerves	Normal nerves
L5 nerve (*n* = 22)	ADC 500	1.9 (± 0.4)	1.7 (± 0.4)
	ADC 800	1.7 (± 0.3)	1.6 (± 0.2)
S1 nerve (*n* = 19)	ADC 500	1.6 (± 0.3)	1.5 (± 0.4)
	ADC 800	1.5 (± 0.3)	1.4 (± 0.2)
S2 nerve (*n* = 7)	ADC 500	1.6 (± 0.2)	1.5 (± 0.3)
	ADC 800	1.5 (± 0.2)	1.4 (± 0.2)
Sciatic (*n* = 4)	ADC 500	1.7 (± 0.2)	1.2 (± 0.2)
	ADC 800	1.5 (± 0.1)	1.2 (± 0.1)

Mean (± SD) of ADC shown by ADC type and anatomical structure, and *p* values from Wilcoxon signed-rank tests were corrected for 8 tests

*Bonferroni-adjusted *p* value < 0.05 when the abnormal nerve was compared to the normal nerve. Non-significant differences determined

**Table 4:** Size measurements (mm) for the abnormal and normal lumbosacral nerves

Anatomical structures	Axis	Abnormal nerves	Normal nerves	Ratio (abnormal/normal)
L5 nerve (*n* = 22)	AP	4.5 (± 1)*	3 (± 1)	1.5 (± 0.4)
	Trans	7 (± 2)*	5.7 (± 1)	1.2 (± 0.3)
S1 nerve (*n* = 19)	AP	4.1 (± 1)*	3.1 (± 1)	1.3 (± 0.3)
	Trans	6.8 (± 1)*	5.4 (± 1)	1.3 (± 0.3)
S2 nerve (*n* = 7)	AP	3.8 (± 1)	2.7 (± 1)	1.4 (± 0.2)
	Trans	6.4 (± 1)	5.7 (± 1)	1.1 (± 0.1)
Sciatic nerve (*n* = 4)	AP	3.3 (± 1)	2.8 (± 1)	1.2 (± 0.1)
	Trans	5.7 (± 1)	4.8 (± 1)	1.2 (± 0.1)

Mean (± SD) of nerve size shown by axis orientation and anatomical structure, and *p* values from Wilcoxon signed-rank tests were corrected for 8 tests

AP = Anteroposterior; Trans = Transverse

*Bonferroni-adjusted *p* value < 0.05 when the abnormal nerve was compared to the normal nerve

**Fig. 6:**
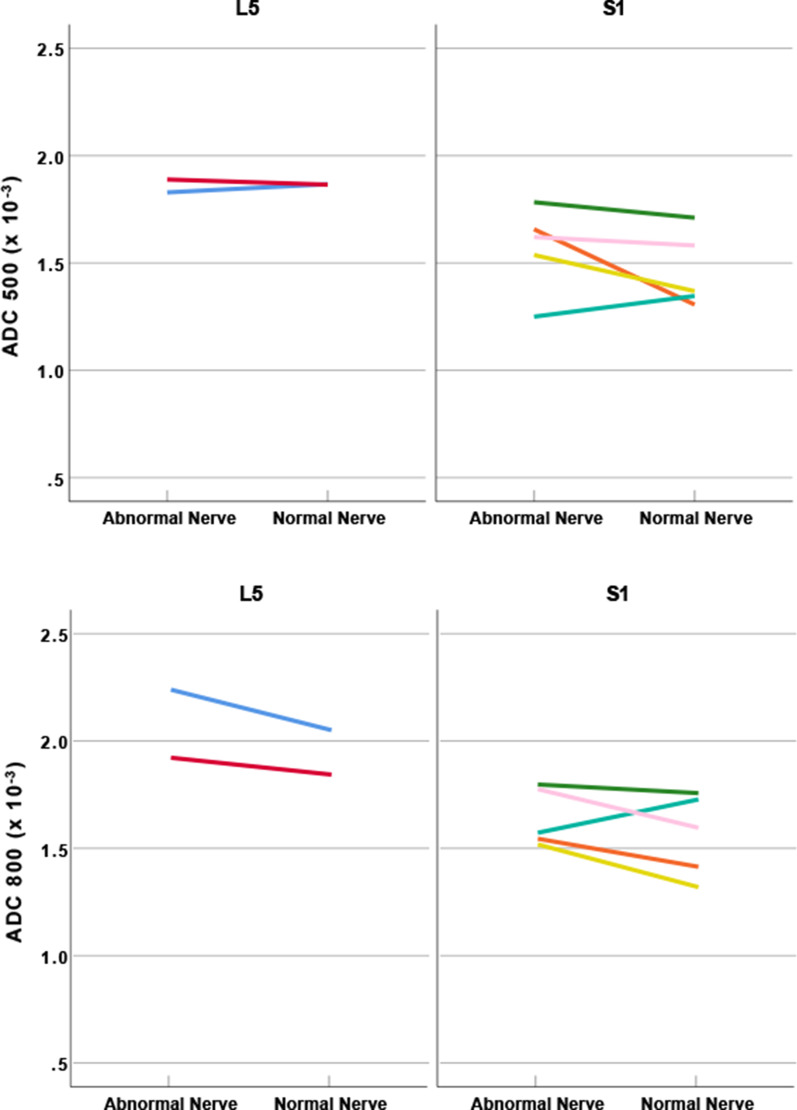
The relationship between ADC values for normal and abnormal lumbosacral nerves. Findings presented are the 7 nerve abnormalities for 7/17 patients who had lumbosacral nerve abnormalities on DWI-RESOLVE which matched both the clinical indications and the EMG/NCV findings. These 7 nerve abnormalities included L5 (*n* = 2) and S1 (*n* = 5). The ADC values at b-500 and b-800 for the abnormal nerves were generally higher than those for the normal nerves. However, in two (*n* = 2) patients, the calculated ADC values for abnormal nerves was lower than for the normal contralateral nerves

The quantitative findings for patients who underwent EMG/NCV testing (*n* = 17), included those for the 7 patients who had lumbosacral nerve abnormalities on DWI-RESOLVE which matched both the clinical indications and the EMG/NCV findings. The quantitative findings for the patients who underwent EMG/NCV testing were comparable to the patients who did not undergo EMG/NCV testing. The measured indices of SNR and ADC were higher for abnormal than normal nerves. While the SNR for normal nerves was lower for the patients who underwent EMG/NCV (Table 5), the ADC values were sometimes higher for normal than abnormal nerves (Fig. 6). Nerve size measurements for EMG/NCV tested patients were greater for abnormal relative to normal nerves in both axes. The size ratio (abnormal/normal) for L5 (*n* = 2) was distributed as follows: 1.6 (± 0.1) and 1.2 (± 0.1) for AP and transverse orientations, respectively, while S1 (*n* = 5) findings were 1.5 (± 0.6) and 1.4 (± 0.1) for AP and transverse orientations, respectively (Table 6).

**Table 5:** Signal-to-noise ratio findings for lumbosacral nerves (EMG/NCV testing patients)

Anatomical structures	Side	DWI-RESOLVE b50	DWI-RESOLVE b500	DWI-RESOLVE b800
L5 nerve (n = 2)	Abnormal	45.2 (± 3.5)	23.7 (± 1.6)	15.3 (± 2.2)
	Normal	31.4 (± 3.6)	16.4 (± 1.7)	11.7 (± 1.4)
S1 nerve (n = 5)	Abnormal	42.9 (± 5.9)	26.7 (± 4.3)	19.9 (± 3.9)
	Normal	30.7 (± 2.8)	19.8 (± 2.4)	15.1 (± 2.7)

Mean (± SD) of SNR shown by sequence type and anatomical structure, and* p* values from Wilcoxon signed-rank tests were corrected for 6 tests

^*^Bonferroni-adjusted *p* value < 0.05 when the abnormal nerve was compared to the normal nerve. Non-significant differences determined

**Table 6:** Size measurements (mm) for the abnormal and normal lumbosacral nerves (EMG/NCV testing patients)

Anatomical structures	Axis	Abnormal nerves	Normal nerves	Ratio (abnormal/normal)
L5 nerve (*n* = 2)	AP	4.5 (± 0.1)	2.7 (± 0.1)	1.6 (± 0.1)
	Trans	6.3 (± 0.1)	5.4 (± 0.2)	1.2 (± 0.1)
S1 nerve (*n* = 5)	AP	3.8 (± 0.1)	2.6 (± 0.1)	1.5 (± 0.6)
	Trans	6.8 (± 0.1)	5 (± 0.1)	1.4 (± 0.1)

Mean (± SD) of nerve size shown by axis orientation and anatomical structure, and *p* values from Wilcoxon signed-rank tests were corrected for 4 tests

AP = Anteroposterior; Trans = Transverse

*Bonferroni-adjusted *p* value < 0.05 when the abnormal nerve was compared to the normal nerve. Non-significant differences determined

## Discussion

Lumbar radiculopathy (or sciatica), defined as pain with possible motor and sensory disturbances in a lumbar nerve-root distribution, is a common symptom with various potential etiologies [1]. A patient’s clinical history and physical examination (e.g., straight-leg-raise test) are only moderately accurate in establishing the diagnosis [22–24]. We have found that the DWI-RESOLVE sequence generated high-quality images of the lumbosacral plexus allowing clear visualization and localization of abnormalities that correlated to patient symptoms. It was determined that affected nerves tend to enlarge and display higher signal intensity relative to normal nerves, which is in-line with previous studies involving the application of EPI DWI at 1.5T [13] and 3T [20].

Currently, EMG/NCV claims to be the most specific technique for evaluating lumbosacral nerves and is regarded as the reference standard in existing literature [25, 26]. Overall, only 36.3% (40/110) of patients with radicular-type symptoms had positive findings on DWI-RESOLVE images and this percentage reasonably matches the EMG/NCV findings (41.2%). This is not unexpected given the difficulty in distinguishing true neural origin radicular symptoms from other etiologies [4]. The inter-observer agreement was excellent for DWI-RESOLVE, indicating that the images reliably demonstrated the abnormality.

For those patients who did not undergo EMG/NCV testing, the quantitative findings generally showed higher mean values for the abnormal compared to normal nerves. As the SNR for normal nerves was lower for the patients who underwent EMG/NCV, the ADC values were sometimes higher for normal than abnormal nerves, which is in line with findings in previous literature [11]. This is potentially the reason for the non-significant differences in the ADC values between both groups.

Although we did not compare different MR neurography sequences, we believe that lumbosacral nerve changes are more evident on DWI-RESOLVE than on other DWI-based sequences previously assessed in the literature e.g., 3D DW steady-state free precession [27] and EPI-DWI [13]. Earlier work has more commonly investigated diffusion tensor imaging (DTI), rather than DWI, for evaluating lumbosacral nerve abnormalities [28–30]. Compared to DWI, DTI images can suffer from loss of directional information when multiple axonal fibers cross within the same voxel [30]. In addition, DTI may result in incomplete tractography tracing, due to isotropic changes and lower fractional anisotropy, which can falsely indicate discontinuity of the nerve [29, 30]. Another limitation of DTI is that it greatly depends on field homogeneity, the gradient and coil system and is thus considered an impractical technique in routine clinical practice [31].

Previous literature evaluated EPI-DWI images with *b* values of 0 and 1000 s/mm^2^ [7, 11, 13]. Our DWI sequence acquired images at *b* values 50, 500 and 800 s/mm^2^. We found these* b* values to be more favorable for the DWI-RESOLVE sequence. On pilot DWI-RESOLVE examinations, we found the SNR of the lumbosacral plexus could be suboptimal at a* b* value of 1000 s/mm^2^ but reasonable at a value of 800 s/mm^2^. While three* b* values were obtained, the b-50 images contributed little to diagnostic accuracy (radiologists did not refer to the b-50 images when assessing signal intensity, and nerve size can be judged adequately from b-500 images). Furthermore, the color maps generated did not require the b-50 images. This suggests a two b-value DWI-RESOLVE sequence (b-500 and b-800 s/mm^2^) may be adequate as this preserves accuracy and reduces scan time.

This adjunct sequence (including b-50 images) added 10 min to a routine lumbar spine MR protocol (7 min if b-50 imaging is not obtained), which is not an insignificant time penalty. However, we consider that the benefits (objective demonstration of a radiculopathy) may outweigh this time cost. While it may be particularly beneficial for patients when the initial clinical diagnosis is clinically uncertain, it may also have a useful role in assessing response to treatment.

A technical limitation of this study is that despite the MR system passing all QA performance tests, magnetic susceptibility artifact was noted on the DWI-RESOLVE images acquired from some patients. This artifact degrades image quality [32], and in particular, adversely affected the quality of the DWI-RESOLVE images of nerves located at the margins of the defined FOV, typically the sciatic nerve. The artifact may obscure the affected nerve or give the false impression of asymmetric nerve signal intensity; however, the artifact is relatively easily identified by radiologists. In this study, nerve evaluation was avoided at the level at which the artifact was identified. The current study showed that the L5 and S1 nerves were principally affected compared with the S2 and/or sciatic nerves, which was in line with a previous study of 97 patients with radicular symptoms which found the number of abnormalities involving the L5 and S1 lumbosacral nerves was higher than for the L4, S2 and S3 lumbosacral nerves [33]. Of note, all the patients characterized as positive with regard to nerve abnormality in this study matched the documented clinical symptoms. In addition, the sample size for the patient cohort with EMG/NCV-confirmed lumbosacral nerves abnormalities was relatively small.

In conclusion, in this study adding a DWI-RESOLVE MR sequence of the lumbosacral plexus to the routine lumbar spine scanning protocol, allowed abnormalities of the lumbosacral nerves to be clearly and accurately demonstrated. This may be useful in objectively confirming a neural cause for radicular symptoms, localizing the cause of symptoms and assessing response to therapies.

## Data Availability

Data is available.

## Abbreviations

ADC: Apparent diffusion coefficient
AP: Anteroposterior
DWI: Diffusion weighted imaging
EMG: Electromyography
MRI: Magnetic resonance imaging
MSK: Musculoskeletal
NCV: Nerve conduction velocity
RESOLVE: Readout segmentation of long variable echo-trains
SI: Signal intensity
SNR: Signal-to-noise ratio
SPAIR: Spectral attenuated inversion recovery
SS-EPI: Single shot echo planar imaging
TSE: Turbo spin echo
